# On the Role of Platelet-Generated Amyloid Beta Peptides in Certain Amyloidosis Health Complications

**DOI:** 10.3389/fimmu.2020.571083

**Published:** 2020-10-02

**Authors:** Mikhail Inyushin, Astrid Zayas-Santiago, Legier Rojas, Lilia Kucheryavykh

**Affiliations:** ^1^ Department of Physiology, Universidad Central del Caribe, Bayamon, Puerto Rico; ^2^ Department of Pathology & Laboratory Medicine, Universidad Central del Caribe, Bayamon, Puerto Rico; ^3^ Department of Biochemistry, Universidad Central del Caribe, Bayamon, Puerto Rico

**Keywords:** amyloid-beta, platelets, Alzheimer’s disease, natural antibiotics, animal models

## Abstract

As do many other immunity-related blood cells, platelets release antimicrobial peptides that kill bacteria, fungi, and even certain viruses. Here we review the literature suggesting that there is a similarity between the antimicrobials released by other blood cells and the amyloid-related Aβ peptide released by platelets. Analyzing the literature, we also propose that platelet-generated Aβ amyloidosis may be more common than currently recognized. This systemic Aβ from a platelet source may participate in various forms of amyloidosis in pathologies ranging from brain cancer, glaucoma, skin Aβ accumulation, and preeclampsia to Alzheimer’s disease and late-stage Parkinson’s disease. We also discuss the advantages and disadvantages of specific animal models for studying platelet-related Aβ. This field is undergoing rapid change, as it evaluates competing ideas in the light of new experimental observations. We summarized both in order to clarify the role of platelet-generated Aβ peptides in amyloidosis-related health disorders, which may be helpful to researchers interested in this growing area of investigation.

## Introduction

Amyloidosis represents a diverse group of diseases characterized by the common factor of deposition of twisted β-pleated sheet fibrils (amyloid) and their aggregates. The β-pleated sheet itself is not abnormal; it is a common motif, usually conserved across species, and a standard secondary structure in proteins, allowing different protein strands (subunits) of a functioning protein to be joined together with hydrogen bonds. The β-pleated sheet forms the basis for uniting subunits in many enzymes and immunoglobulins, as well as channel-forming subunits of specific ion channels and pores. Formation of β-pleated sheet hydrogen bonds between two or more parallel protein strands requires standard spacing between amino acids in these parallel polypeptides. It also requires correct subunit assembly and the right organization of the process (1). Unfortunately, this bond formation between parallel chains may occur pathologically because of mutations augmenting the binding propensity of particular polypeptides or the elevated concentration or overproduction of specific peptide chains, allowing the formation of polymeric ß-pleated sheets consisting mainly of multiple copies of the same type of chain. This interaction causes the proteins to form misfolded pathologic polymers, usually fibrils and aggregates, in a process called amyloidosis. The different forms of amyloidosis are classified by the composition of the amyloid fibrils and the manner of their deposition, which may be local or systemic. In amyloid light-chain (AL) amyloidosis (also known as primary amyloidosis, as it is the most common form), the free light chain of the immunoglobulin molecule (termed in clinical practice the Bence Jones protein) is hyper-secreted by lymphocyte cells in blood plasma. In many cases, it is linked to cancer) (2, 3). While in the immunoglobulin fold, on which the β-sheet formation is healthy, the high concentration of only the light chain makes this process abnormal (2, 4). The accumulation of AL amyloid, which can be local or systemic, disrupts the tissue architecture and, in conjunction with a toxic effect from the oligomeric light chains (5), leads to severe organ damage that may involve the kidneys, heart, liver, peripheral nerves, and even bones. Systemic amyloidosis (which can be of the senile type or an early-onset familial type) is the result of the deposition of transthyretin (TTR) protein. TTR is a serum and cerebrospinal fluid carrier known for its transport of retinol, the thyroid pre-hormone thyroxine (T4), and also some peptides. It usually circulates as a homo-tetramer, but, due to genetic mutation, tetramers can dissociate into monomers that then misassemble into amyloid fibrils (6). In their senile form, TTR monomers become fragmented and mix with full-size monomers, leading to misfolded aggregates (7). Reactive systemic amyloidosis is the result of an overproduction of a non-immunoglobulin protein, AA, which is associated with blood serum. There can also be amyloidosis related to the overproduction of β2 microglobulin (B2M amyloidosis), a free protein with an antibacterial activity that is a light chain of the major histocompatibility complex protein (8). The production of amyloidogenic proteins in all the abovementioned forms of amyloidosis directly originates in blood cells or is related to blood plasma. Generally speaking, the depositions, in many cases, spread from the blood to inside the organs, with the highest concentration around blood vessels. In previously described types of amyloidosis, blood vessel damage is also common (9, 10).

Alzheimer’s disease (AD) is the only well-known form of severe amyloidosis in which the amyloidogenic peptide is believed to be produced in organ tissue and not systemically in blood plasma. The main component of amyloid fibrils and other amyloid aggregates in AD are the amyloid beta (Aβ) peptides. Another common component of these aggregates is the amyloid P component (AP), a normal blood plasma constituent (11) produced by the liver, and its concentration in blood plasma has been shown to be about five-fold elevated in AD (12).

This exclusive association of brain tissue with the production of materials that form plaques in AD may be explained historically. Amyloid cerebrovascular senile plaques were described by Dr. Alois Alzheimer in the brain of dementia patients a century ago, and it was found later that these plaques contain Aβ peptides (13), while both neurons and astrocytes can produce these peptides (14). In addition, animal models that used neuron-associated promoters to generate the aggregation-prone mutated Aβ had shown many similarities in morphology and pathophysiology with the brains of AD patients [for review see: (15)]. This mechanism was therefore extrapolated to late-onset AD. Recently, multiple findings have emerged suggesting that there may be a flow of Aβ from blood to the brain in AD in which platelets are vital players [reviewed in (16)]. Platelets were also suggested as the most important source of Aβ in glaucoma [reviewed in (17)]. In this review, we used the Web of Science, PubMed, and Google patent databases to search for studies examining the role of systemic release of Aβ in a variety of health complications that exhibit Aβ accumulation of oligomers or plaque deposition. We ask related questions that have not been discussed in previous reviews and discuss the advantages and disadvantages of existing animal models for studying platelet-related Aβ in AD and other diseases.

## Aβ Peptide Accumulation Is Associated With A Variety Of Diseases

Aβ peptides may be of varying length (<46 amino acids) and have a specific sequence, which differs only slightly across mammalian species (18). Due to hydrogen bonding between the parallel monomers, Aβ peptides are prone to form dimeric, tetrameric, or higher-order oligomers, even at very low concentrations (µm range), while at higher concentrations they associate into filaments that tend to join in misfolded aggregations known as amyloid plaques (19–21). The presence of Aβ extracellular plaques suggest that the concentrations of Aβ are elevated in the affected tissues. However, Aβ aggregation can start at lower concentrations due to specific mutations within Aβ and its precursor. For example, such mutations are the basis of hereditary early-onset familial AD (22). Aβ peptides of different lengths also have different propensities to aggregate (15), and the amyloidogenic properties of Aβ peptides from humans and other mammals may be different. For example, the propensity of murine Aβ to produce insoluble amyloid aggregations is limited (23), (also see below), and the majority of murine transgenic AD models involve the expression of mutated human Aβ.

However, besides AD, a variety of health problems have, as a common component, the accumulation of Aβ in tissues at elevated concentrations, sometimes leading to its aggregation. It was discovered that plasma levels of Aβ peptides in pancreatic, as well as in esophageal, colorectal, hepatic, and lung cancer patients were significantly higher than in healthy controls (24, 25). In glioblastoma, Aβ was found in both oligomeric and aggregated forms to be associated with glioma cells as well as localized in the tumor extracellular space, and it was proposed that blood could be the source of this peptide (26, 27). It was shown that platelets are activated near cancer tumors, playing the role of “first responders” during cancer development and metastasis (28).

Aβ is elevated near blood vessels and forms transient amyloid plaques in the zone of traumatic brain injury or stroke (29–35). It was proposed that Aβ accumulation in astrocytes and on blood vessel walls is related to ischemia in these processes, while both brain cells (30) and platelets (35–38) can be the source of Aβ. Using immunocytochemistry, we detected a massive release of Aβ peptides in and around blood vessels in the brain and skin after experimental thrombosis, and we determined the source of these peptides to be platelets (39, 40). Interestingly, according to evidence in the literature, murine Aβ deposits are transient after traumatic brain injury, while in humans, they are relatively stable.

Aβ peptides also accumulate in the myocardium with ischemic heart failure, while circulating levels of Aβ are predictive of cardiovascular mortality in patients with coronary heart disease (41, 42). The sources of Aβ involved in this process are still not known, but we propose that Aβ generated from a platelet precursor could be at least one of these sources.

Aβ (and other amyloidogenic proteins) also accumulate in the placenta during preeclampsia, a leading contributor to maternal and perinatal morbidity and mortality worldwide. There are malformations to placental blood vessels in this condition. The attempt of the body to compensate these malformations probably leads to extremely high blood pressure. This induces vessel damage and inflammation in the placenta, leading to local amyloid accumulation, including Aβ (43). This condition usually produces hemolysis and affects blood composition (44).

During glaucoma, Aβ accumulates in the retina, mainly within the layer of apoptotic retinal ganglion cells (RGC) near the region of microvascular changes in the eye. During this disease, the rearrangement of damaged blood vessels occurs in the zone of the entrance of blood vessels and the optic nerve into the retina, producing anatomic changes, termed cupping. Aβ released in this area thus may be the cause of retinal cell death, previously associated only with the effects of high intraocular pressure (17, 44–46). It was found that application of synthetic Aβ induces significant RGC apoptosis *in vivo*, while anti-Aβ treatment was effective in the prevention of RGC apoptosis in glaucoma patients (17, 47–51). Additionally, some anti-glaucoma medicines have apparent anti-platelet effects, suggesting that platelets participate in glaucoma development (52).

Also, accumulation of Aβ is evident in the advanced stages of Parkinson’s disease (PD) (53–55). While PD motor impairment, which develops due to α-synucleinopathy and dopamine deficiency, is devastating, later progressive cognitive impairment and dementia (PDD) eventually become the major debilitating symptoms for 80% of PD patients, and these have no cure (54, 56). From the early stages, after α-synucleinopathy advances in PD patients, Aβ becomes visible in the brain as well (57), and after 20 years approximately 50% of PDD patients develop extensive neuropathologies similar to AD. These include misfolded Aβ plaques and tau neurofibrillary tangles, mainly in the frontal cortex and striatum (58, 59), while the scale of Aβ-produced damage and its effects on PDD development are still being debated (54, 55, 57, 60–62). It was also found that there is an accumulation of insoluble Aβ around blood vessels (cerebral amyloid angiopathy, CAA) in 53% of PD patients (63). In sporadic AD, striatal depositions are rare (but common in early-onset AD, (64), while they are predominant in PD and PDD. Although the striatum and frontal cortex are the zones of massive degeneration of the neuronal processes of dopamine neurons as well as inflammation in PD (65), it is still difficult to differentiate the role of Aβ in “pure” AD from PD with Aβ depositions and to determine the source of these depositions in PD.

Here it should be remarked that, while it is known that Aβ peptides in humans can be of different lengths, with different properties, reported measurements of the Aβ40/Aβ42 ratio in many pathologies (except AD) are unfortunately rare, and we will not discuss this issue here. Moreover, the buildup of extracellular plaques due to Aβ aggregation occurs in brain tissue, in the vicinity of skin blood vessels, or in peripheral blood vessels in internal organs (40, 66). Most likely, it is related to the difference in blood vessel wall structure in these areas and in other parts of the body. It is known that brain blood vessels and peripheral blood vessels have a size barrier formed by the inter-endothelial junctions (IEJs) between endothelial cells (67, 68). This junction barrier defines paracellular permeability, not allowing phagocytes to enter the nearby tissue and producing a “no-cleanup” zone in brain and around peripheral blood vessels, shifting the balance between accumulation and removal of extracellular plaques.

There are other health conditions in which the occurrence of Aβ oligomers, fibrils, and plaques are common (16). Nevertheless, the best-studied disease related to Aβ is AD.

## Aβ In Alzheimer’s Disease

Aβ was found to be the major component of amyloid depositions described in the brain of AD patients (13), while Aβ oligomers at high concentrations probably ignite the disease itself (15, 21). Aβ oligomers damage neurons, inducing tangle formation. Neuronal tangles start to appear (those that correlate with brain impairment) when amyloid concentration is high, and greater concentrations of Aβ oligomers and amyloid plaques correlate with tangle spread (69).

While Aβ deposition in AD was discovered first in the brain, deposits or high concentrations of oligomers of Aβ were later described in peripheral tissues during the course of this disease. It can be found in the skin, certain muscles, heart tissue, the eye (in the retina and the lens), and even the intestines of patients (66, 70–73).

The presence of Aβ aggregates locally or systemically during many health problems, together with the known antibiotic activity of Aβ (see below), led many researchers to suggest that hyperproduction of Aβ is a typical defensive reaction of innate immunity (16, 17). The generation and release of Aβ in large quantities (hyperproduction) in pathological cases results in its aggregation and accumulation as a side effect of this response. The ultimate cause of the disease can be various infections or mechanical damage that activates this systemic release of Aβ. Released for protection against multiple invasions, Aβ later becomes the damaging factor for the tissue, creating a positive feedback in the vicious cycle of the disease. The question arises: where is the systemic production of Aβ concentrated, and how does it work?

## Aβ Is An Innate Immunity Weapon Released By Platelets

### Aβ Is an Antibiotic Agent

Aβ peptides have strong antibiotic activity against both Gram-negative and Gram-positive bacteria, as well as fungi and viruses (74–77). Aβ also combats mouse microbial infections *in vivo* (78). Extracellular entrapment of the invading agent may be one of the mechanisms of this antibiotic effect. As an example, it was shown that certain defensins, peptides produced by neutrophils and certain other blood cells, have a propensity to arrange themselves in amyloids. For instance, human α-defensin 6 forms ß-pleated sheet fibrils with antimicrobial properties entangling the bacteria in net-like structures (79, 80). Similarly, it was shown that Aβ peptide oligomers aggregated into fibrils entrap microbes (78) or can bind herpes virus surface glycoproteins, accelerating Aβ deposition and leading to protective viral entrapment (81). Other defensins can form large, weakly anion-selective ion channels, and this channel-forming ability contributes to their antimicrobial properties (82). Equally, we have shown that a synthetic Aβ peptide perforates the external membrane of yeast (40), and it is known that natural peptide antibiotics with channel-forming activity kill target cells, including fungi, by this same mechanism (83, 84). It was shown earlier that soluble Aβ peptide oligomers at low concentrations perforate cell membranes by forming tetrameric/octameric channels penetrable by K^+^ ions, while at higher concentrations they form large, non-selective pores (85–89). An excess of Ca^++^ permeability through these pores induces calcium dyshomeostasis and is extremely toxic (90, 91). Large pores also allow large molecules entry into the cell. Based on these findings, it has been suggested that, like defensins, Aβ is a previously unrecognized antimicrobial agent that usually functions in the innate immune system (16, 38, 75, 78, 92). Other researchers and our group believe that Aβ may be released as a response to infection (16, 81), and this release is likely triggered by tissue damage and inflammation (17, 40).

### Platelets Are the Primary Source of Systemic APP and Aβ

Amyloid beta (Aβ) peptides may be of various lengths (<46 amino acids) but have a specifically conserved sequence, with 90% similarity between vertebrate species but still with significant differences [see (18)]. These peptides are produced by a two-step (ß+γ) cleavage from a longer amyloid precursor protein (APP), a process occurring in many cell types, for example in neurons and astrocytes in the brain (15). This APP processing is known as the amyloidogenic pathway, because it produces Aβ and is enhanced during pathology; for example, it was found to occur in AD (93), while the same APP is processed differently (the non-amyloidogenic pathway) under normal physiological conditions. Due to hydrogen bonding between parallel monomers, Aβ may form dimeric, tetrameric, or higher-order oligomers, even at very low concentrations. At higher concentrations, it associates into larger β-pleated sheets, forming filaments tending to join in misfolded aggregations known as amyloid plaques (19, 20). The buildup of extracellular plaques in AD and other conditions (e.g., brain trauma and cancer) suggests that the concentration of Aβ is elevated in an affected individual’s tissue. Aβ aggregation can start at a lower concentration, due to specific mutations within Aβ and its precursor that augment the propensity of Aβ peptides to aggregate, forming the basis for hereditary early-onset familial AD (22). Our group and others have already reviewed the literature on the possible sources of Aβ in AD and certain other diseases (16, 46, 94), and it has been suggested that there is significant local production of Aβ by neurons and probably astrocytes and that APP processing can be found in the brain and enteric nervous system (15, 95, 96). There is strong evidence that cultured neurons may produce Aβ and even form “plaques in the dish” (16). Multiple AD murine transgene models with human mutant Aβ generated in neurons under the control of specific neuronal promoters have shown important characteristics of AD, such as extracellular amyloid plaques, cerebral amyloid angiopathy (CAA), and sequential development of tauopathy (97–99). Although none of the animal models fully replicates the human disease, they have contributed essential insights into the pathophysiology of Aβ biology and toxicity.

However, there is another systemic source of APP and Aβ: platelets, which are small nuclear cells formed from the pro-platelet processes of the megakaryocyte (MK) precursor cell (100, 101). While MK cells originate in the bone marrow, and many researchers believe that platelets also originate there (102), it has been shown that at least 50% of platelets are generated from megakaryocyte-type extravascular progenitors in the pulmonary capillary bed of the lungs at the site of high oxygen tension (103–106). Platelet production from MK cells is tightly regulated by diverse humoral factors (100, 101). Platelets contain various types of granules, including α-granules, dense granules, and lysosomes (107). Besides coagulation factors, platelet α-granules contain APP, which is expressed predominantly as two isoforms of increasing length (751 and 770 amino acids), both containing a Kunitz proteinase inhibitor (KPI) domain (108, 109). APP can be liberated upon platelet degranulation (110–115) and represents about half of all protein secreted from agonist-treated platelets (111). APP with a Kunitz-type protease inhibitor can effectively inhibit chymotrypsin, trypsin, and other proteolytic enzymes (111, 116) and promotes activation of coagulation factor XII, affecting the hemostasis and temporal stability of the thrombus (117, 118). Platelets may also generate Aβ peptides and are the primary source (~90%) of this peptide in human blood (119). While APP processing in platelets under normal physiological conditions is mostly non-amyloidogenic, it changes during the response to pathology. Investigators studying AD biomarkers used platelets to examine the components of both the non-amyloidogenic and amyloidogenic cascades, finding that platelets are an excellent model with which to study blood-based AD-related biomarkers, reflecting a shift in Aβ production during AD (120). It was previously suggested that whether platelets generate soluble APP or either of the Aβ peptides is determined by a specialized regulated secretory vesicle pathway (121, 122) different from any found in neurons. In either setting, APP or its cleavage products are released mainly within extracellular vesicles, although with a different type of γ-secretase and localization of APP during the two-step (ß+γ) cleavage:

(1) In its neuronal secretory pathway, APP is always a type 1 transmembrane protein and is located in the membrane. First, cleavage of APP by β-secretase occurs in a soluble environment, while secondary cleavage by γ-secretase occurs within the transmembrane domain of the APP when inserted into the membrane, thus liberating Aβ outside the cell or inside certain cellular vesicles (123, 124). In neurons, γ-secretase is a proteolytic complex consisting of four proteins. Presenilin (PS) is the active core, while the other three proteins provide support functions (125). In neurons, Aβ is released at nerve terminals in the CNS after the precursor APP is transported there by axonal transport (126, 127). Cleavage processing most probably occurs in a type of endosome known as a multi-vesicular body (MVB) in the terminals, the intracellular structures that contain smaller vesicles released from the cell in the form of exosomes when the MVB fuses with the plasma membrane (128, 129). These exosomes contain mainly APP cleavage products and have a variety of receptors reacting with nearby neurons and astrocytes (130).

(2) In the secretory pathway, vesicles may release both full-length, soluble APP, and/or Aβ. This event is known to occur in platelets (110, 111) and in chromaffin cells (131), and both cell types have specialized secretory vesicles. Full-length APP within vesicles exists mainly in its soluble form. It has β- and γ-secretase sites accessible for proteolytic cleavage inside the vesicle’s soluble environment, thereby also releasing Aβ inside the vesicle lumen (131). The content of vesicles is released by the cell in a regulated process, and it may be APP that is released, or APP may be processed further inside the vesicle. In platelets, α-granules represent the final evolution of MVBs and contain exosomes, similar to the MVBs in neurons (132). The α-granule content can be extruded or fused to the external membrane (133), liberating exosomes, as also occurs in neurons. The cathepsin B and D enzymes, which can cleave soluble APP, are described as a β-secretases in this pathway. It was suggested that this regulated secretory pathway (121, 122, 134) produces the major portion of secreted, extracellular Aβ peptides.

It was also found that macrophages may engulf platelets and process APP to produce Aβ in atherosclerosis (135). In addition, brain vessel endothelial cell enzymes can cleave the platelet-released APP, forming Aβ, most efficiently if the activated platelets adhere directly to the endothelial cells (136). Leukocytes can also produce and release Aβ, but the amounts are small relative to that produced by platelets (137). Similarly, many other cells, such as fibroblasts and endothelial cells, may produce small amounts of Aβ (138). Summarizing, we can say that platelet-generated Aβ may be a significant component of systemic Aβ. Now the question arises: what is the role of systemic Aβ?

## Aβ Is A Vital Defense Protein With Multiple Roles

Evolutionarily, mammalian platelets became denucleated and reduced in size to small (1–2 µm) cells, thereby having a high surface-to-volume ratio that accelerated the speed of reception and granule secretion, with the further ability to easily transit from tissue to blood and back through gaps between endothelial cells everywhere except in the brain. These advantages made them useful as first responders, which are most important in hemostasis and innate immunity. In this review, we are primarily focused on the link between tissue damage and inflammation and the generation of platelet-associated Aβ peptides. Many comorbid bacteria and viruses were found in patient brains during AD or glaucoma (17). We suggest that Aβ peptides can be generated from APP released by platelets in response to inflammation of septic, mechanical, or chemical origin.

### Aβ Is Generated by Platelets During Coagulation

We used immunostaining to visualize Aβ after photothrombosis in mouse brains and found that, upon coagulation, the increased concentration of platelets allows enhanced release of Aβ. Aβ immunostaining was intense inside and near blood vessels in the thrombotic zone, with the maximum intensity near the vessel walls (39). Similarly, Aβ generated from precursors released from platelets might be the source of its accumulation in mouse skin, as it was found to be concentrated around blood vessels after experimental thrombosis (40). A similar accumulation of Aβ around blood vessels in the skin of AD patients and generally in older patients was described many years ago (66, 139). Moreover, we recently reported that Aβ immunofluorescence accumulated on blood vessel walls in the damaged part of the brain and on nearby astrocytes after middle cerebral artery occlusion (35). Temporary accumulation of Aβ in GFAP-positive astrocytic bodies and processes that formed clusters with specific small vessel-like structures was reported previously (29, 140–143), see also review: (38). Aβ-containing plaques, as determined by immunofluorescence, but not plaques staining positive for Congo red or thioflavin (aggregation-specific amyloid stains) can persist for up to 9 months after arterial occlusion (144). Also, temporary Aβ plaques appeared in the brain of an AD mouse model after mild brain trauma. They then disappeared after 7 days, which was correlated with the post-traumatic concentration of soluble Aβ oligomers in the brain (145). Aβ plaques and oligomers may also be found in the brains of human patients within hours of traumatic brain injury (TBI) in non-AD patients (33, 146, 147). These findings, taken together, suggest that trauma followed by coagulation is an important cause of Aβ accumulation in tissues.

### Platelets in the Immune Response

It is known that platelets act as important mediators of innate defenses: platelet adhesion, activation, and degranulation are the essential steps in this process, in which platelet-associated surface receptor molecules play a pivotal role in the development of inflammation (148).

Platelets express CD40L and toll-like receptors (TLR), which recognize microbe-associated threats and may modulate innate immunity or directly interact with microorganisms and viruses (17, 149–152). Platelets can engulf bacteria and viruses in endosome‐like vacuoles that fuse with α‐granules with antimicrobial contents (153). When directly activated by viral and bacterial antigens, platelets release microbicidal peptides (16, 154–162). We have shown that Aβ peptides perforate yeast cell membranes while not affecting somatic cell membranes at the same concentration (40). Apart from Aβ peptide, there are other antibacterial peptides released by platelets. Like Aβ, one of these antibacterial peptides from rabbit platelets is cleaved from a longer precursor and has a variable length of 72–73 amino acids (159).

Moreover, platelets 1) interact with other immune cells using cell-specific adhesion molecules, 2) attach themselves to neutrophils and monocytes at the site of lesion and also activate these cells as well as themselves, 3) release multiple antibacterial factors, and 4) participate in both innate and acquired immune responses (163, 164). In addition, platelets have close interactions with the innate complement system, while being protected themselves from complement-mediated damage by soluble and membrane-expressed complement regulators. Still, they also bind several complement components on their surface and trigger complement activation in the fluid phase (165). The best-studied mechanism is the joint work of platelets and neutrophils in forming circulating platelet–neutrophil complexes: stimulation of the neutrophil surface receptor TLR type 2 (TLR2) amplifies the release of α‐granules and membrane expression of P-selectin on the surface of platelets. P-selectin allows adhesive interactions with leukocytes and endothelial cells *via* P-selectin glycoprotein ligand 1, which activates leukocyte production of cytokine cascades and initiates or further promotes inflammation (166). At the same time, platelets promote the recruitment of neutrophils to sites of tissue damage. They bind with activated neutrophils and endothelial cells on vessel walls, forming platelet–neutrophil aggregations and stimulating the production of filamentous neutrophil extracellular traps (NETs), which trap and kill pathogens (132, 151, 167–170). It has been shown that aggregated platelets at high density secrete mainly Aβ peptides ending at residue 40 (Aβ40) as a final product, while the Aβ42 level is not affected by cell density (171).

**Figure 1:**
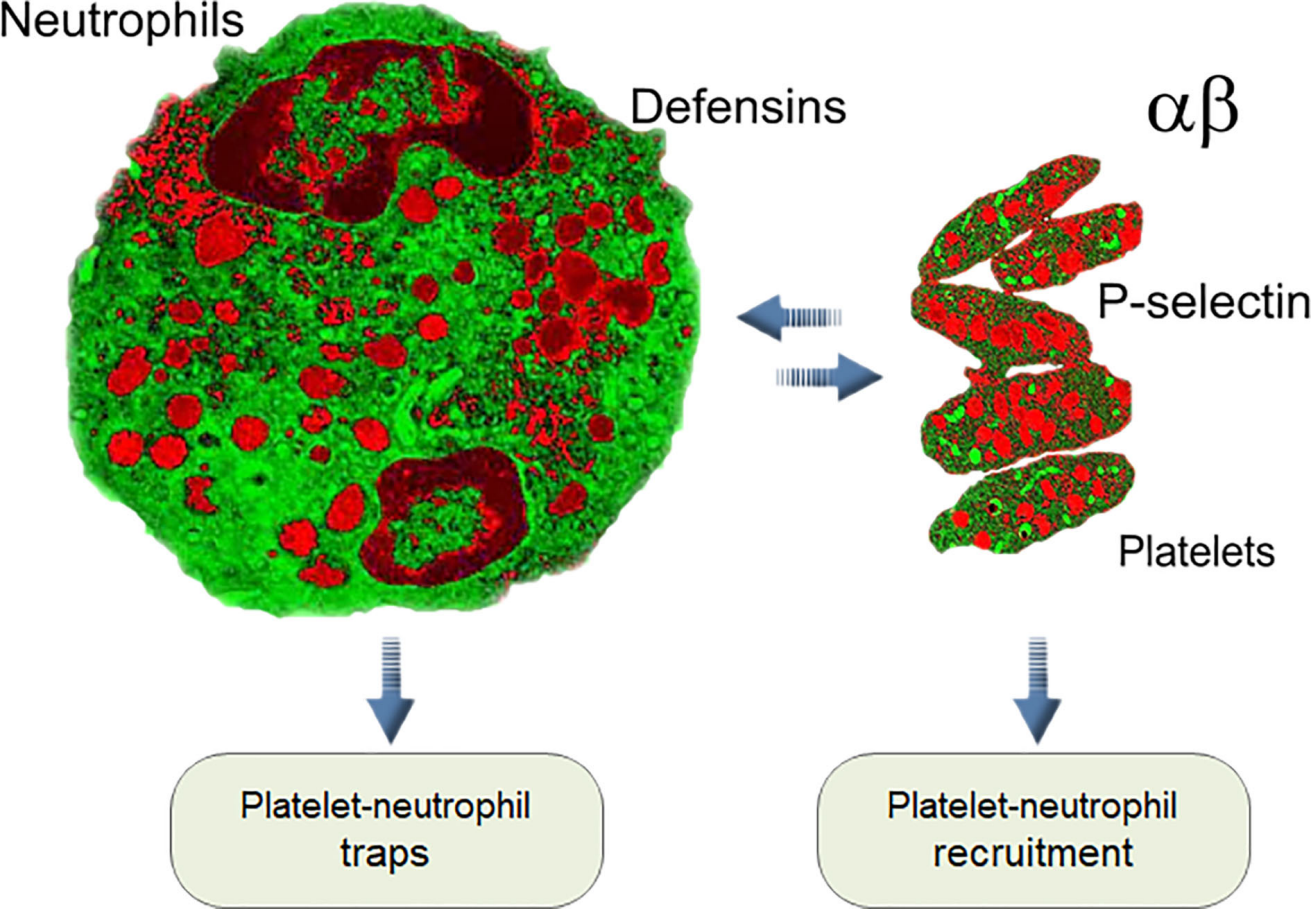
Interaction between platelets and neutrophils is one of the key elements of innate immunity in mammals (see text).

Additionally, an unusual reverse influence of neutrophils on platelets, known as emperipolesis, was reported. In this process megakaryocytes engulf neutrophils, fusing with their membranes and subsequently producing “daughter” platelets containing neutrophil membrane and membrane receptors. The entire process of emperipolesis takes a few minutes, after which the neutrophil liberates itself and egresses intact from the megakaryocyte. This process enables neutrophils passing through the megakaryocyte cytoplasm to modulate the production and membrane content of platelets (172). All these interactions between neutrophils and platelets in normal blood and during infection, inflammation, and thrombosis are the pillars of the immune–hemostatic continuum [**Figure 1**, (166, 173–175)]. The connection between neutrophils and platelets led us to compare their antimicrobial arsenals, and they showed striking similarities.

### The Similarities Between Aβ Peptides and Defensins

While there are a variety of mammalian defensins, all are synthesized as a larger precursor molecule and then cleaved a varying number of times to obtain the final product. They are active against bacteria, fungi, and many different viruses. For example, human neutrophil peptides (HNP)-1–3 are first synthesized as the 94-amino-acid (aa) preproHNP, which is converted to 75-aa proHNPs by cotranslational removal of a 19-aa endoplasmic reticulum signal peptide. At the promyelocytic stage of myelopoiesis, proHNPs are further cleaved and accumulate in azurophil granules in neutrophils as 29–30-aa HNPs. By contrast, the proHNPs produced by more mature myeloid cells undergo a high degree of constitutive exocytosis without cleavage. These prodefensins have no antimicrobial potential, and the significance of their secretion is unknown (176, 177). Antimicrobial action is mediated *via* several mechanisms, including pore formation or aggregation. For example, the antimicrobial peptide human defensin 6 (HD6) can aggregate, forming amyloid filaments with a strong affinity for bacterial surfaces and thereby trapping bacteria (69). By contrast, (HNP)-1–3 at low concentrations form a lipophilic β-sheet-rich dimer with additional disulfide bonding, but at higher concentrations they can oligomerize into tetramers, hexamers, and larger oligomers, creating a variety of pores or less-well‐defined apertures, termed “giant aggregate channels,” in plasma membranes, thereby killing cells (178).

Aβ peptides, while relatively short, are synthesized as longer (680–780 aa) APPs. Then, like defensins, the APPs are cleaved twice (with β- and γ-secretases) to obtain a final length of 36–43 aa for the mature Aβ peptide. They are also active against bacteria, fungi, and many different viruses, and their antimicrobial action is mediated *via* several mechanisms, including pore formation and aggregation. Soluble Aβ peptide oligomers at low concentrations (50–200 nM) perforate cell membranes by forming tetrameric channels penetrable by K^+^ ions and do so at higher concentrations by creating Ca^++^-permeable hexameric pores, while they may also form large pores (86–88). The main toxic effect that has been suggested is related to the excess Ca^++^ permeability through these pores, which induces calcium dyshomeostasis (90, 91). Other toxic agents may also enter the membrane aperture to kill the cell (179). In our experiments, the external membrane of the yeast was perforated by synthetic Aβ at a 5-mM concentration (40). A similar range of concentrations (10–40 µM) was shown for synthetic defensin-forming channels in fungal membranes (180). We also suggest that the effective concentration of peptides (lipophilic defensins and Aβ) for pore formation can be much lower if they are solubilized with selective carriers, such as transthyretin or apolipoproteins. Recently, it was shown that certain external compounds that react with Aβ might modulate its effects by working as carriers (181).

It is known that small and double-bridging peptides are resistant to many proteases, tolerating digestion, even following oral administration (182). A structure with four sulfide bridges and multiple β-strands linked to an α-helix is typical of defensins, making them resistant to proteases. Additionally, certain defensins have antipeptidase activity themselves or may regulate secretory leukocyte protease inhibitor α2 macroglobulin, which allows them to block microbial proteases with synergistic combinations of defensin and protease inhibitor (183) but also allows them to resist host proteases.

Aβ oligomers usually lack disulfide bridges, except for certain mutant peptides (184), but they have multiple β-strands reinforced with salt bridges (185). Besides, in many cases Aβ peptides are released jointly with a full-size APP or its fragments with Kunitz-type domains, which block protease activity and protect the released Aβ peptide. It was shown that the amount of released Kunitz-APP is vital for AD development and is correlated with the number of neurotic plaques (186).

It is common knowledge that Aβ concentration is augmented in AD and certain other conditions, but the same is true for defensins. Rapid accumulation of defensins proximal to the site of brain inflammation occurs with neurodegeneration (187), including in AD (188), bacterial and viral infection, and brain trauma (188–190). Antimicrobial peptide β-defensin-1 expression is also upregulated in AD brain, especially in the choroid plexus but also in astrocytes and blood vessel walls (191, 192). Under physiological conditions, dendritic cells are restricted to the meninges and choroid plexus of the brain and are generally not present within the brain parenchyma (193). In addition, there are several antimicrobial peptides with a clear structural resemblance to defensins, with similar pore-forming and mesh-forming activities [for a review see: (194)].

## A Possible Rodent Model Of Platelet-Generated Aβ

Studies of platelet-generated Aβ must reproduce the following effects: 1) induced APP is expressed in platelets; 2) platelet-generated Aβ is prone to aggregation; 3) platelet-generated Aβ can be transported from the blood to the brain or some other tissue of interest, as some Aβ mutants are not transportable.

### Expressing an APP of Interest in Platelets Using Different Promoters

The expression of Aβ in a transgenic model depends on the type of promoter used to control its expression. Different promoters have a stably recurring expression in specific cells, while some have remarkable variation in expression patterns (195). Of the most common promotors used in mouse transgenes, the prion promoter element (PrP) is most promising. It is mainly active in brain neurons but also in extraneuronal regions, especially in cells with secretory granules (196). It was found that exosomes release cellular prion protein from activated platelets (197, 198). Similarly, APP was found to be concentrated in exosomes of a specific size in platelets (199). This gives hope that a transgene with an inserted variant of APP and under control of the PrP promoter can generate both APP and Aß in association with platelets as well as with neurons.

Another promising promoter is the rat platelet factor 4 promoter element (rPF4). A transgenic mouse that generated modest overexpression of induced human wild type APP (770 isoforms) in platelets was constructed (200). However, in this animal model, mouse and not human Aß was found in the brain (201), raising the possibility that human wild type Aß has a transport impediment at the blood–brain barrier (BBB) in mice.

The popular mouse Thy 1.1 promoter is used in many murine transgenes that develop Aβ accumulation in brains of mouse and rat and CAA-type aggregation in blood vessels (202, 203). However, this promoter does not transcribe well in platelets and is usually manipulated (by intron 3 deletion) to remove its transcription in cells other than neurons (204, 205). Therefore, platelets have no expression of transgenic APP, but express only endogenous wild type APP. There are reports that truncated Thy 1 can also be activated in endothelial cells by inflammation (206). Interestingly, blood vessel damage in organotypic wild type brain slices was ascribed to platelets because of their platelet-generated Aβ (207). Platelets were harvested from Tg-SwDI mice with APP expressed under a Thy 1 promoter, and therefore we suggest that Aß in platelets from these animals was mainly wild-type and not transgenic. Kniewallner et al. showed that these AD-derived platelets more aggressively damage healthy vessels in any case and that matrix metalloproteinase hyperactivation was involved. Thus, even wild-type platelet-generated Aß can produce damage if platelets are hyperactivated.

Summarizing, the majority of murine transgenic models of AD use the insertion of mutated human APP variants, and many of these transgenes do not express human Aβ in platelets. This must be taken into account when evaluating platelet-related studies of Aβ accumulation.

### Aggregation of Generated Aβ and Transit Barriers

It is known that Aβ wild type and variants have different tendencies to aggregate. Human Aβ(1–40) and Aβ(1–42) differ in their ability to form amyloid fibrils (208), while it was also shown that both variants can co-aggregate, creating mixed β-sheets (209). In addition, there is a species-related difference: the propensity of murine Aβ to produce amyloid deposits is limited, even in aged mice. This is because human and murine APPs differ at three amino acid residues within the Aβ peptide sequence and are cleaved differently by β-site APP cleaving enzyme 1 (BASE1), thereby producing mainly shortened Aβ fragments not prone to aggregation or easily soluble aggregates in wild type rodents (23, 210). Therefore, practically all transgenic mouse models of AD amyloid deposition use somewhat humanized APP. It can be a mutated human APP or a murine APP that is chimerized to include human-type early-onset mutations to generate Aβ deposits. Human presenilin (a component of the cleaving mechanism) must be added to produce longer Aβ peptides. For example, when expressed in mouse APP695, a transgene with mutations resembling Swedish human mutations leading to early-onset AD (APPswe) and reinforced by a human presenilin exon-9-deletion variant (PS1dE9) can produce amyloid deposits consisting entirely of mouse Aβ peptides that are morphologically similar to deposits found in humans during early-onset AD (211). Recently, using a parabiosis procedure on this APPswe/PS1dE9 transgenic AD mouse with their wild-type littermates, it was directly established that human Aβ originating from the transgenic AD mouse model entered the circulation, accumulated in the brains of the wild-type mice, and formed cerebral amyloid angiopathy and Aβ plaques after 12 months of parabiosis (212). The authors did not determine the source of blood-derived Aβ but suggested that the source may be platelets. This chimerical mouse/human amyloid precursor protein (Mo/HuAPP695swe), together with mutant human presenilin 1 (PS1-dE9), was directed to CNS neurons and platelets with a PrP promoter. It is possible that the Aβ in this model first penetrated the BBB from the brain of the transgenic mouse and then once again the BBB of the littermate, passing through this barrier twice. Alternatively, Aβ may simply be transported from platelets in the circulation to the littermate brain. In any case, at least one BBB transit mechanism was involved. The same mouse model (APPswe/PS1dE9) was used to show that thrombotic cerebrovascular lesions induce a rapid transient increase in amyloid plaque burden and amyloid angiopathy in the area immediately surrounding the infarcted area, (213). These and other results suggest that this model (APPswe/PS1dE9) is the best for studying the effects of platelet-generated Aβ.

Another interesting problem is hybrid aggregation. Wild type Aβ from one cell type and a mutant Aβ from neurons may aggregate, forming hybrid (hetero-)oligomers, thus affecting amyloid formation. For example, if a heterozygote animal has two different Aβ variants, one variant could reduce self-assembly of the fibrils of the other variant. Some Aβ mutants even have opposite parallel or antiparallel β-sheet arrangements in oligomers [as was shown for the Italian E22K and Iowa D23N mutations; (214)]. It is known that shorter Aβ fragments can aggregate with full-length Aβ, and the resulting oligomers will block self-assembly of the fibrils and amyloid (215). Thus, wild type Aβ fragments from platelets being transported to the brain may interfere with fibril formation by mutant Aβ from a neuronal source in transgenic animals. Are Aβ peptides transported to the brain and back? Fragments of mutant and hybrid Aβ oligomers may have transit barriers at the BBB, and this possibility has been largely unstudied.

Aβ may be transported in and out of the brain parenchyma by several physiological mechanisms. The vascular luminal receptor for advanced glycation end products (RAGE) is thought to be a primary transporter of Aβ across the BBB into the brain from the systemic circulation. The low-density lipoprotein receptor-related protein (LRP)-1 (expressed mainly at the abluminal side of the BBB) mediates transport of Aβ out of the brain (216–219).

The Italian E22K and Iowa D23N mutations can result in the formation of Aβ oligomers and fibrils, with an antiparallel β-sheet structure predisposing them to be deposited in cerebral blood vessels rather than accumulating mainly in plaques through distinct interactions with the receptors responsible for Aβ clearance across the BBB (214). As already mentioned, human Aβ probably encounters a transit barrier in murine models. For example, poor clearance of human Dutch/Iowa mutant Aβ40 peptides from mouse and rat brain was shown (203, 220). This factor may also be important for studying platelet-generated amyloid peptides in murine models.

## Conclusions

- There are a number of health complications in which high levels of Aβ peptides and Aβ amyloid aggregates occur.
- While many cells may produce Aβ, including neurons and astrocytes, platelets are the primary source of systemic APP and Aβ.
- Platelets are a vital part of intrinsic immunity, and Aβ is an essential defense protein released during trauma and coagulation and as a response to inflammation. Aβ has evident antimicrobial and antiviral properties, suggesting that inflammation-related tissue accumulation of Aβ may be an overreaction against microbial or other aseptic causes.
- Platelets are essential players in tissue Aβ accumulation in AD, glioma, and glaucoma and may be involved in other neurodegenerative diseases, such as PD.
- While the direct release of APP and its non-amyloidogenic products is prevalent in platelets under normal physiological conditions, our literature review suggests that, in many pathologies, platelet activity shifts to Aβ production and that inflammation is one of the triggers.
- The propensities of Aβ from different animal species and humans to aggregate are different, and murine Aβ does not form stable aggregates. Thus, the majority of murine transgenic models of AD use the insertion of mutated human APP variants, and many of these transgenes do not express human Aβ in platelets. This must be considered when interpreting the results of platelet-related studies of Aβ accumulation. Some human Aβ may also encounter a transport filter at the mouse blood–brain barrier.

## Author Contributions

MI, AZ-S, LR, and LK reviewed the literature and wrote this review; MI and LR prepared the figure.

## Funding

NIH NIGMS SC2GM111149 grant supported MI during this work.

## Conflict of Interest

The authors declare that the research was conducted in the absence of any commercial or financial relationships that could be construed as a potential conflict of interest.
